# Seed Morphology in *Silene* Based on Geometric Models

**DOI:** 10.3390/plants9121787

**Published:** 2020-12-16

**Authors:** José Javier Martín-Gómez, Agnieszka Rewicz, José Luis Rodríguez-Lorenzo, Bohuslav Janoušek, Emilio Cervantes

**Affiliations:** 1 IRNASA-CSIC, Cordel de Merinas 40, 37008 Salamanca, Spain; jjavier.martin@irnasa.csic.es; 2 Department of Biogeography, Paleoecology and Nature Conservation, Faculty of Biology and Environmental Protection, University of Lodz, 1/3 Banacha Str., 90-237 Lodz, Poland; agnieszka.rewicz@biol.uni.lodz.pl; 3 Plant Developmental Genetics, Institute of Biophysics v.v.i, Academy of Sciences of the Czech Republic, Královopolská 135, 612 65 Brno, Czech Republic; rodriguez@ibp.cz (J.L.R.-L.); janousek@ibp.cz (B.J.)

**Keywords:** cardioid, geometric models, seed morphology, seed shape, taxonomy

## Abstract

Seed description in morphology is often based on adjectives such as “spherical”, “globular”, or “reniform”, but this does not provide a quantitative method. A new morphological approach based on the comparison of seed images with geometric models provides a seed description in *Silene* species on a quantitative basis. The novelty of the proposed method is based in the comparison of the seed images with geometric models according to a cardioid shape. The *J* index is a measurement that indicates the seed percentage of similarity with a cardioid or cardioid-derived figures used as models. The seeds of *Silene* species have high values of similarity with the cardioid and cardioid-derived models (*J* index superior to 90). The comparison with different figures allows species description and differentiation. The method is applied here to seeds of 21 species and models are proposed for some of them including *S. diclinis*, an endangered species. The method is discussed in the context of previous comparison with the measures used in traditional morphometric analysis. The similarity of seed images with geometric figures opens a new perspective for the automatized taxonomical evaluation of samples linking seed morphology to functional traits in endangered *Silene* species.

## 1. Introduction

The Caryophyllaceae Juss. (Caryophyllales Jussieu ex Bercht. and J. Presl) comprises about 90 genera and 2625 species, of a wide distribution with their greatest diversity in temperate climate [1,2,3,4]. *Silene* L. is the largest genus of the Caryophyllaceae [5]. The number of species in the genus varies between taxonomic treatments, including from 700 [6,7] to 800–900 species [4,8]. Most of them are diploid (2n = 2x = 24) but there are also tetraploid, hexaploid and octoploid species [9]. Species of *Silene* are annual, biennial, and perennial herbs distributed mainly across the Northern Hemisphere with two main centers of diversity: The South Balkan Peninsula and South-West of Asia [6,7,9]. At least 12 endangered species have been reported in *Silene*, including five in Spain: *S. diclinis* (Lag.) M. Laínz [10], *S. fernandezii* Jeanm., *S. gazulensis* A.Galán de Mera, J.E. Cortés, J.A. Vicente Orellana and R. Morales Alonso, S. *hifacensis* (Rouy ex Willk.) O. Bolòs and Vigo [11,12], and *S. sennenii* Pau [13]. According to comprehensive gene tree analyses based on the nrDNA ITS and cpDNA rps16 and phylogenetic tree analysis including 262 samples representing 243 species, the genus *Silene* has been split in three subgenera: *S.* subg. *Lychnis*, *S.* subg. *Behenantha*, and *Silene sensu stricto* [14]. The authors of this article already indicated the lack of a morphological diagnostic key in support of this distribution, due to a high degree of homoplasy.

Seed morphology provides important information in taxonomy, and it has been applied to genera in the Caryophyllaceae, such as *Arenaria* L. [15,16], *Gypsophila* L. [17,18], *Moehringia* L. [19], *Paronychia* Mill. [20], *Sagina* L. [21], *Stellaria* L. [22], and *Velezia* L. [23]. *Silene* is the best studied genus in the Caryophyllaceae in terms of seed morphology [5,24,25,26,27,28,29,30,31].

Seed morphology studies in *Silene* have a long tradition and present descriptions of seed shape and ornamentation. In 1869, Rohrbach applied to *Silene* seeds the expression *reniformia* (kidney-shaped), and introduced a classification based on the structure of the back of the seed as plane (*dorso plana*) or deepened (*dorso canaliculata*). His research sought the importance of seed morphology in the description of *Silene* seeds indicating: ”*denn in der That bietet die Gestalt des Samens das sicherste Kennzeichen zur Unterscheidung sehr vieler species*” (because in fact the shape of the seed offers the most reliable indicator for the differentiation of many species) [25]. Variation was observed in size and shape as well as in seed coat microstructure and the structure of lateral and dorsal faces [27,28,29,30,31]. Hoseini et al., 2017 described species and sections keeping common traits not only macromorphologically but also in the microstructure of the seed [31].

Since Rohrbach’s work, seed shape description in *Silene* species is based on adjectives such as “reniform”, “circular”, “globular”, or “semi-globular” [6,9,29,30]. However, these adjectives are not precise because they refer simultaneously to two different characters: two-dimensional images of the seed and the three-dimensional shape. For example, the term “reniform” means that the overall structure resembles a kidney, and “globular” means that it resembles a sphere. In the first case, it is not possible to have quantitative data because the kidney is not a geometrically defined figure, and in both cases, there are no described means to determine the degree of similarity to a kidney or to quantify sphericity of the seeds. To address this situation, we present a method based on the comparison of the seed images with bi-dimensional geometric figures described mathematically [32,33]. This allows the quantification of the two-dimensional shape of the seeds by the percentage of similarity between the seed image and a given geometric figure. This measure was termed *J* index. The method was first applied to *Arabidopsis thaliana* (L.) Heynh. [34] and later to the model legumes *Lotus japonicus* L. and *Medicago truncatula* Gaertn. [35,36] among other species.

**Figure 1:**
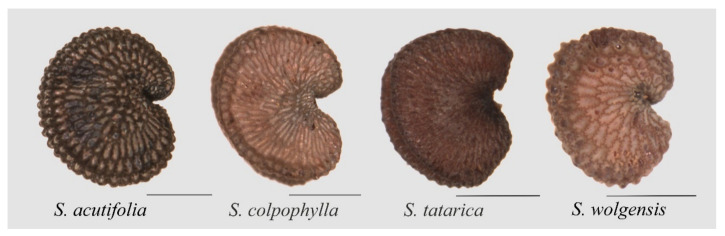
Seed asymmetry is frequently observed in *S. acutifolia*, *S. colpophylla*, *S. tatarica,* and *S. wolgensis*. Bar represents 0.5 mm.

The bidimensional images of the seeds of *Arabidopsis thaliana* resemble a cardioid elongated by a factor of *Phi* (The Golden Ratio = 1.618) in the horizontal axis [34], while the seeds of *Medicago truncatula* resemble a cardioid elongated by a factor of *Phi* in the vertical axis [35], and *Lotus japonicus* seeds adjust well to a cardioid [35,36]. Also, the seeds of *Capparis spinosa* L. adjust well to the cardioid [37], as well as species of the Papaveraceae and Malvaceae [38,39]. Ovals and ellipses were the models for seed shape quantification in many species of the Cucurbitaceae [40] and the Euphorbiaceae, such as *Ricinus* L. and *Jatropha* L. [41,42]. The description of shape in wheat kernels was based on three geometric figures: (1) an ellipse of aspect ratio (AR) = 1.8 for the “round varieties” (*Triticum aestivum* subsp. *aestivum* var. Zebra and Torka), (2) a lens of AR = 3.2 for the elongated kernels *(T. monococcum* L.), and (3) an ellipse of AR = 2.4 with the intermediate-shaped varieties such as *T. turgidum* subsp. *durum* cv. Floradur [43]. The seeds of species in the Vitaceae, as well as diverse cultivars of *Vitis vinifera*, were accurately described with a set of morphological models based on heart-shaped and piriform curves [44]. Studies of comparative morphology in *Silene* species based on geometric models can provide original information about taxonomic relationships pointing as well to associations between seed shape and ecological properties.

The main objective of this work is to define, for the first time, geometric models adjusting the bi-dimensional images of the seeds of species from the genus *Silene*, including the threatened species *S. diclinis* and assess their value in taxonomic classification. In addition, other morphological aspects are described and their application in classification is discussed.

## 2. Results

### 2.1. General Morphological Description: Size and Shape in the Seeds of Silene Species

**Figure A1:**
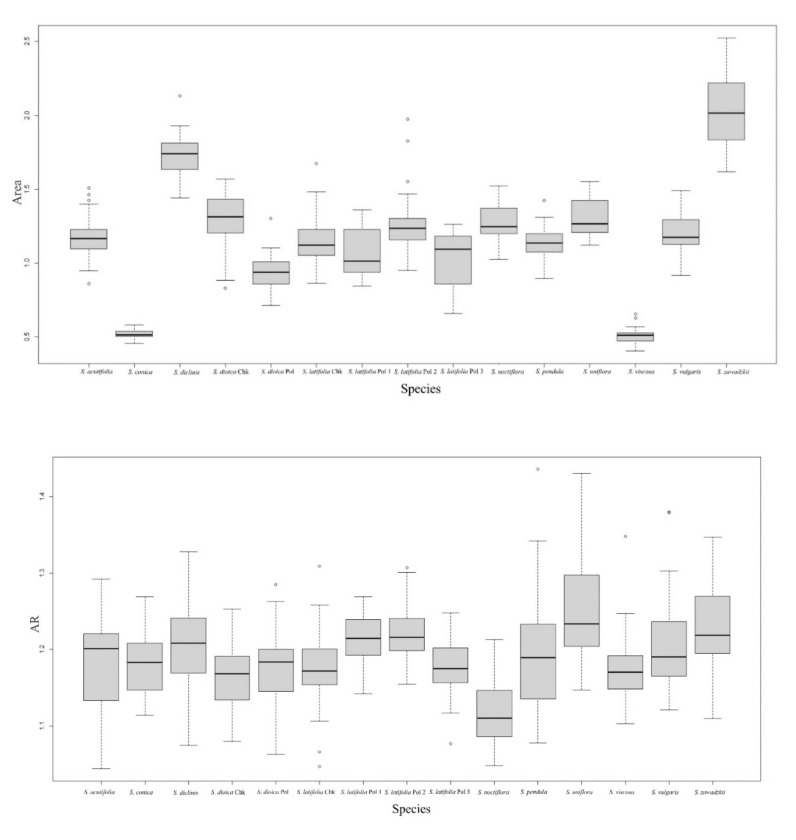

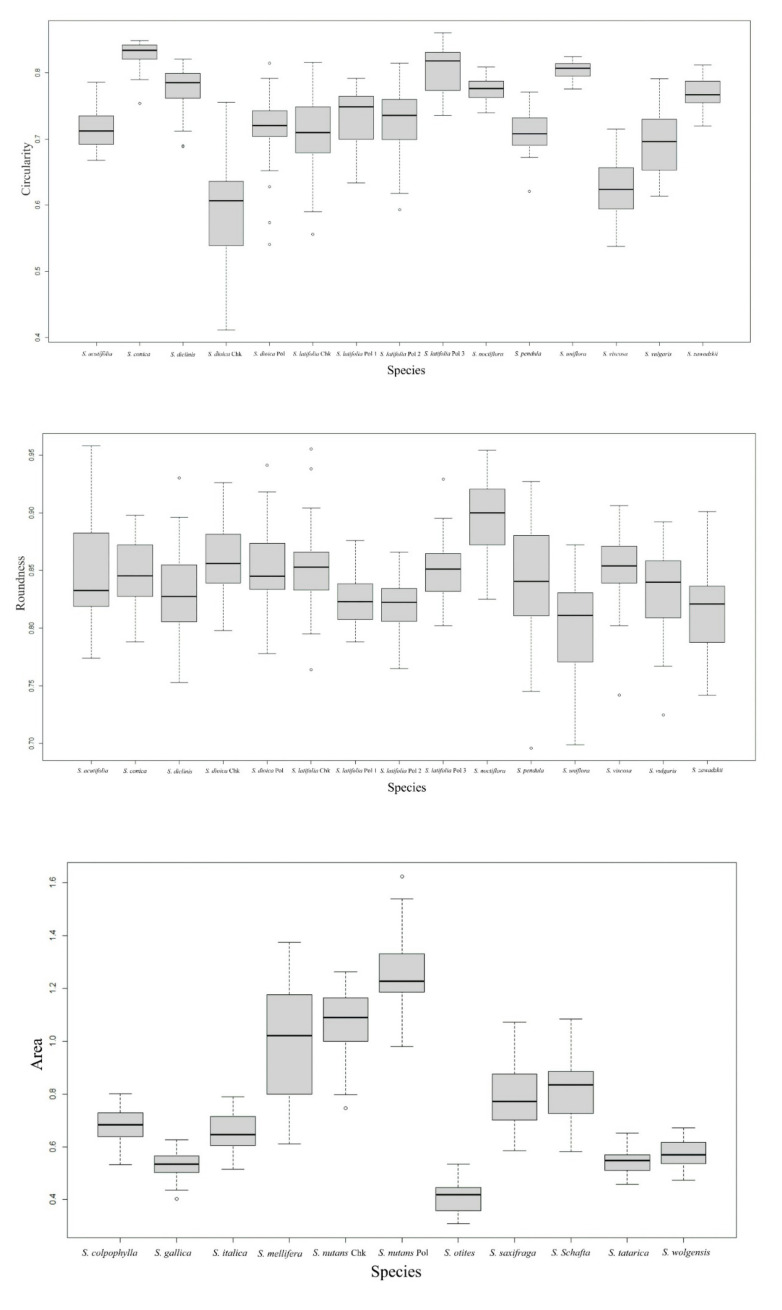

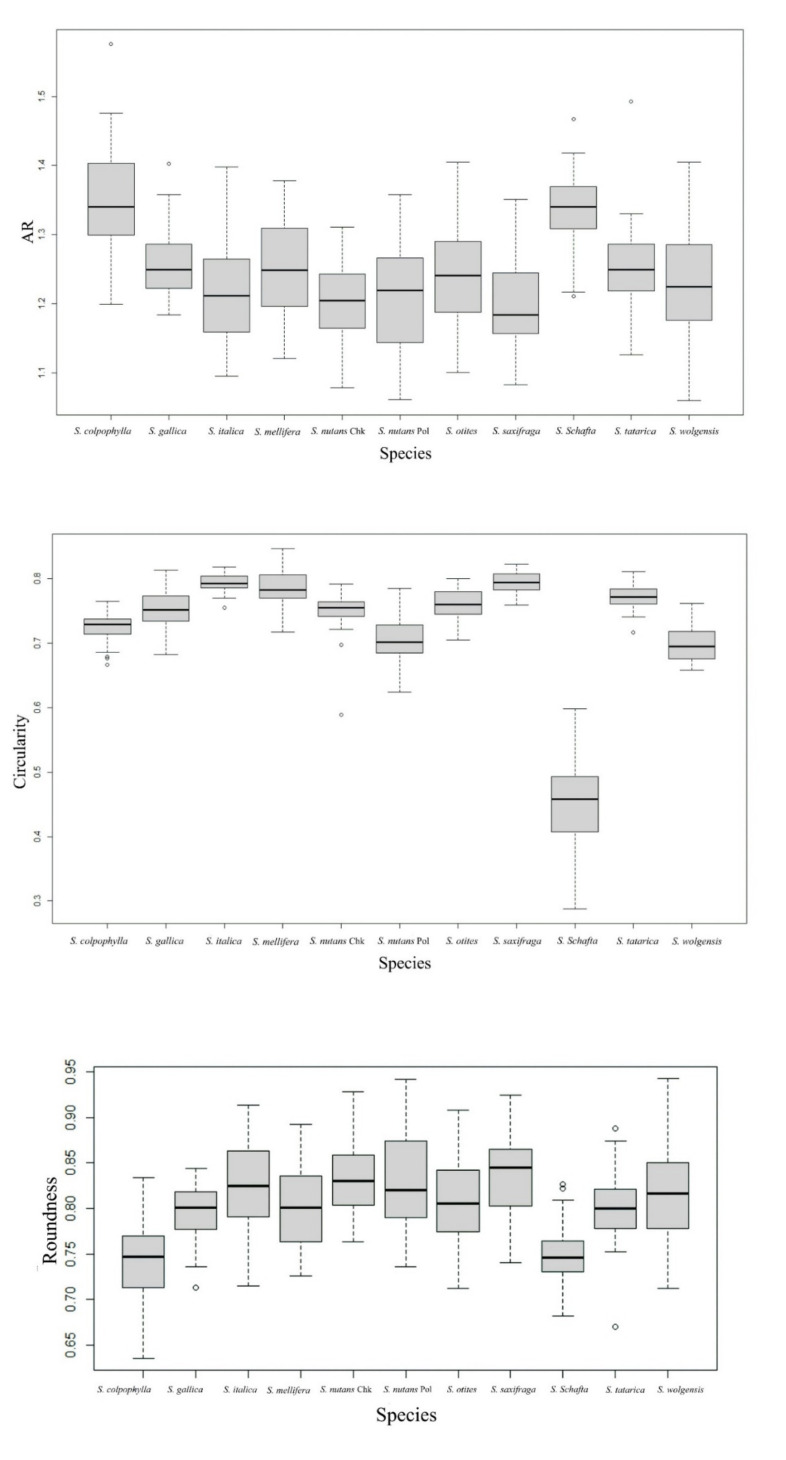
Box plot representations corresponding to four selected measurements (Area, Aspect Ratio, Circularity and Roundness), for *S*. subg. *Behenantha* and *S*. subg. *Silene* respectively. From left to right in all images of *S*. subg. *Behenantha* (first four images): *S. acutifolia, S. conica, S. diclinis, S. dioica* Chk, *S. dioica* Pol, *S. latifolia* Chk, *S. latifolia* Pol 1, *S. latifolia* Pol 2, *S. latifolia* Pol 2, *S. noctiflora, S. pendula, S. uniflora, S. viscosa, S. vulgaris, S. zawadskii.* From left to right in all images of *S*. subg. *Silene* (second group of four images): *S. colpophylla, S. gallica, S. italica, S. mellifera, S. nutans* Chk, *S. nutans* Pol, *S. otites, S. saxifraga, S. schafta, S. tatarica, S. wolgensis*. Upper and lower limits of the discontinuous lines represent the maximum and minimum values not atypical (atypical values are outside, below the discontinuous lines). Lower and upper limits of the boxes represent respectively the first and third quartile. The thickened bar in the box is the median.

Table 1 and Table 2 present a general morphological description of the seeds in groups corresponding to *S*. subg. *Behenantha* and *S*. subg. *Silene*. Table 1 contains data for eleven species belonging to S. subg. *Behenantha* and Table 2 presents the data corresponding to ten species of *S*. subg. *Silene*. Figure A1 contains the box plots corresponding to four selected characters (Area, Aspect Ratio, Circularity, and Roundness), for *S*. subg. *Behenantha* and *S*. subg. *Silene* respectively.

Notable differences between species were found for all parameters analyzed. In the species of S. subg. *Behenantha*, seed image area was comprised between 0.51 mm^2^ in *S. viscosa* and 2.02 mm^2^ in *S. zawadzkii*. These values were extreme, with most species having mean area values comprised between 1.10 and 1.73 mm^2^. The two stocks of *S. dioica*, one in the collection of Poland and the other in Czech Republic, had different values in A, P, L, W, and C, but not in AR or R. Differences between stocks of *S. latifolia* were observed in all measurements.

The aspect ratio was comprised between 1.12 and 1.25 with an opposed trend to both circularity and roundness, and roundness values were higher than those of circularity. In cases where seed surface has prolongations increasing seed perimeter, circularity is reduced while roundness maintains high values. The values of roundness were higher in *S. noctiflora* (0.90) than in the other species (comprised between 0.80 and 0.86). Circularity values were comprised between 0.59 (*S. dioica* Chk) and 0.83 in *S. conica*.

In the species of *S*. subg. *Silene*, seed image area was comprised between 0.41 mm^2^ in *S. otites* and 1.07 mm^2^ in *S. nutans* (Table 2). The values of aspect ratio were between 1.20 (*S. saxifraga*) and 1.35 (*S. colpophylla*), while the circularity was particularly low in *S. schafta*, due to its surface protuberances, but it was comprised between 0.70 and 0.79 in the other species. Roundness values were comprised between 0.74 (*S. colpophylla*) and 0.84 (*S. saxifraga*). In both, the species of *S*. subg. *Behenantha* and the species of *S*. subg. *Silene*, values of aspect ratio, circularity and roundness had lower variation rates than the measurements of size.

### 2.2. Structural Aspects

Structural aspects include seed surface and other properties of seeds, including asymmetry, the presence of a dorsal face and the existence of a ridge. 

#### 2.2.1. Seed Surface

**Figure A2:**
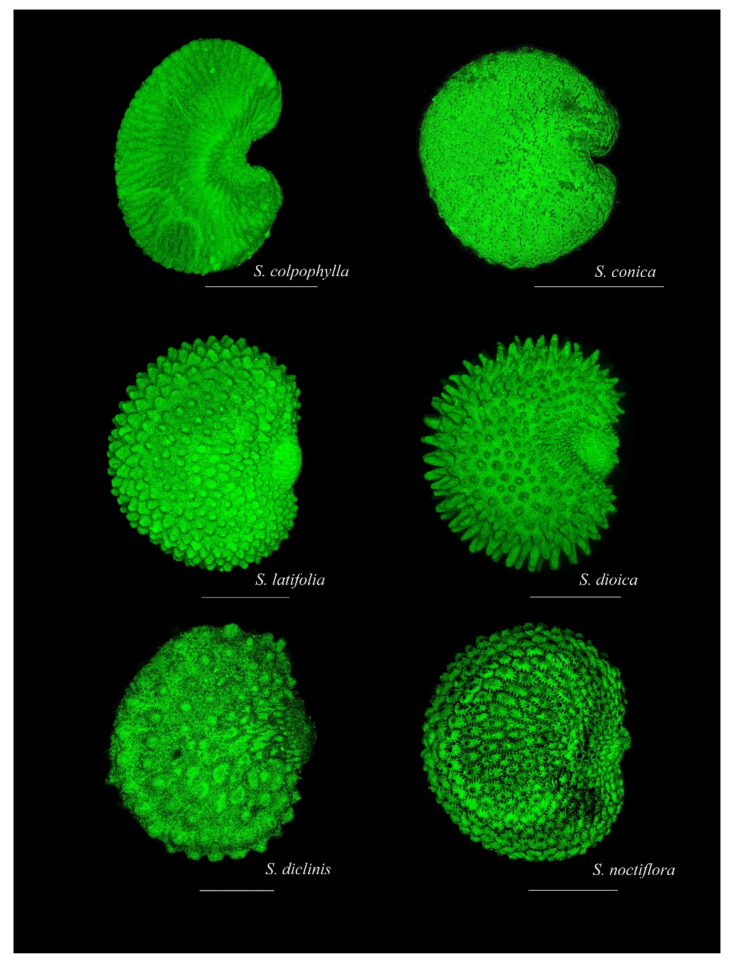
Confocal microscopy images of *Silene* seeds. Each figure contains the mean projection of a series of 20 images. Seed surface in *S. colpophylla* and *S. conica* (above) is smooth. In contrast, other species present colliculae. The colliculae may be conical-obtuse (*S. latifolia*), conical-acute (*S. dioica*) or rounded like in *S. diclinis* and *S. noctiflora* (below). Bars represent 0.5 mm.

Surface texture is better appreciated in confocal microscopy (Figure A2). The seed surface can be smooth, such as in *S. colpophylla* and *S. conica* or covered by colliculae, that may be conical (acute) resulting in a radiate structure (*S. dioica*), conical-obtuse (*S. gallica*, *S. latifolia*) or rounded (*S. acutifolia*, *S. diclinis*, *S. noctiflora*, and *S. nutans*).

#### 2.2.2. Other Properties of Seeds

In addition to surface structure, other aspects in the morphological description of *Silene* seeds include: (1) asymmetry, (2) a pronounced dorsal face that may be plane or concave, and (3) presence of a ridge. Table 3 presents a summary of the variability in the morphological characteristics of the species studied.

**Figure 2:**
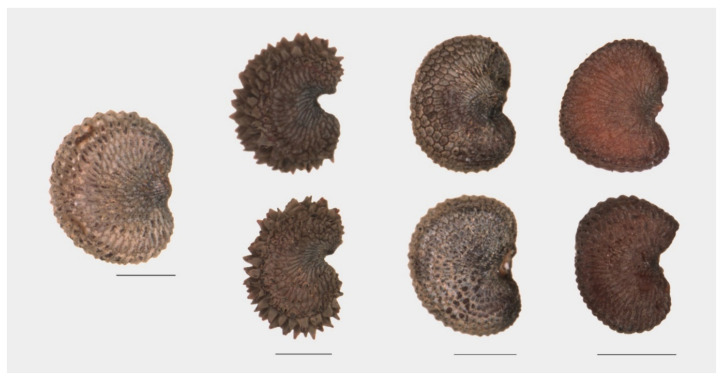
Types of asymmetry observed in *Silene* species. Left: a symmetric seed (*S. nutans*). Next, left to right: Type 1: *S. schafta* (the two lobes of different size and/or shape); Type 2: *S. mellifera* (one lobe rounded and the other flattened); Type 3: *S. tatarica* (one lobe rounded and the other flattened and inclined). Bars represent 0.5 mm.

A marked asymmetry is observed in the seeds of *S. acutifolia, S. colpophylla, S. tatarica,* and *S. wolgensis* (Figure 1). In all the images used, the seeds are oriented with the micropile to the right so that approximately half of the seed is above a hypothetical horizontal line passing through the micropile, and the other half is below this line. The asymmetry consists of differences above and below the micropyle. Three types can be described: (1) Asymmetric seeds with the two lobes more or less rounded and of different size and/or shape; (2) asymmetric seeds with one rounded lobe and the other flattened, and vertical or almost vertical; and (3) asymmetric seeds with one rounded lobe and the other flattened and inclined. Figure 2 contains examples of these types of asymmetry.

Asymmetric seeds are more frequent in species of *S*. subg. *Silene* than in *S*. subg. *Behenantha* (Table 2).

**Figure 3:**
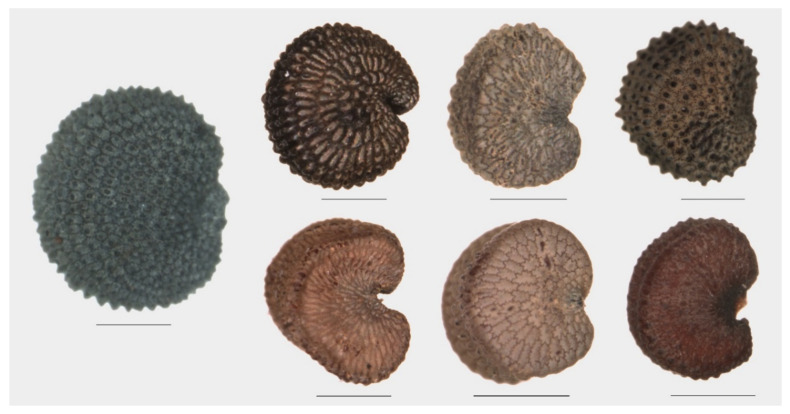
Representative examples of seeds with their dorsal face plane and canaliculate. Left: *S. dioica* as a control (dorsal face rounded, no particular structures). Above, three examples of dorsal face plane: *S. acutifolia*, *S. italica*, and *S. pendula*. Below, three examples of dorsal face canaliculate: *S. colpophylla*, *S. conica*, and *S. tatarica*. Bars represent 0.5 mm.

Seeds of some species have a dorsal structure that can be plane or concave, forming a channel (*S. colpophyla*, *S. conica*) corresponding to the two classes that were termed by Rohrbach as dorso-plana and dorso-canaliculata [25] (p. 50) (Figure 3). These types of dorsal structure are more frequent in the seeds of species of *S.* subg. *Silene* than in *S.* subg. *Behenantha* (Table 2).

**Figure 4:**
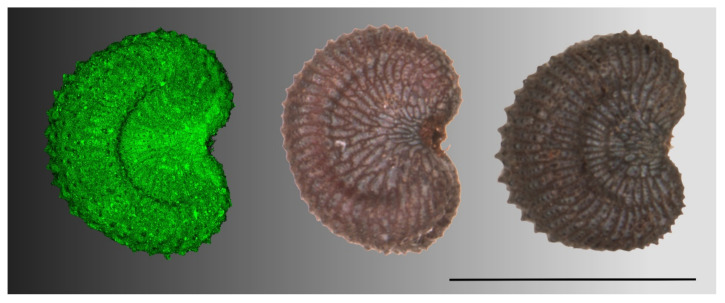
Seeds of *S. gallica* present a conspicuous ridge. Bar represents 1 mm.

The seeds of *S. gallica* present a conspicuous ridge (Figure 4). This structure is also observed in other species, such as *S. nutans*, *S. uniflora,* and *S. zawadzkii*, but in a lower number of seeds and with a lesser definition than in *S. gallica*.

### 2.3. Morphological Description of Silene Species by Similarity with Geometric Models

The morphological description includes the analysis of similarity with the cardioid, a comparison between subgenera and the analysis of similarity with other figures related to the cardioid in some species. 

#### 2.3.1. Similarity with the Cardioid. *J* Index Values

**Figure 5:**
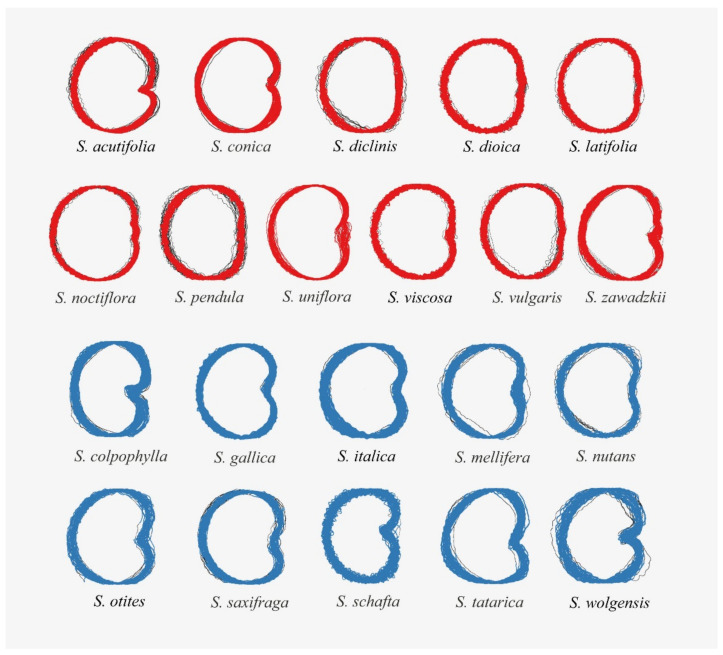
The silhouettes of 40 seeds of each of the species of *Silene* grouped according to subgenus (*S*. subg. *Behenantha* in red; *S*. subg. *Silene* in blue). The seed silhouettes have been scaled to fit a common proportional size without affecting shape.

**Figure 6:**
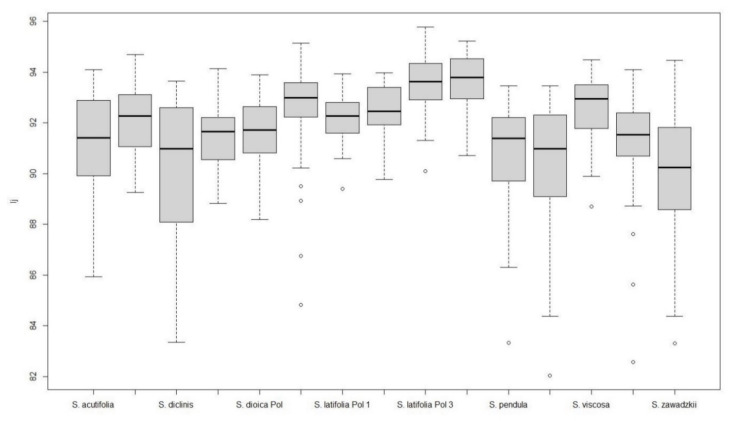
Box plot representing the values of *J* index with Model 1 for species of *S*. subg. *Behenantha*. Left to right: *S. acutifolia*, *S. conica*, *S. diclinis*, *S. dioica* Chk, *S. dioica* Pol, *S. latifolia* Chk, *S. latifolia* Pol 1, *S. latifolia* Pol 2, *S. latifolia* Pol 2, *S. noctiflora*, *S. pendula*, *S. uniflora*, *S. viscosa*, *S. vulgaris*, *S. zawadskii*. Upper and lower limits of the discontinuous lines represent the maximum and minimum values not atypical (atypical values are outside, below the discontinuous lines). Lower and upper limits of the boxes represent respectively the first and third quartile. The thickened bar in the box is the median.

**Figure 7:**
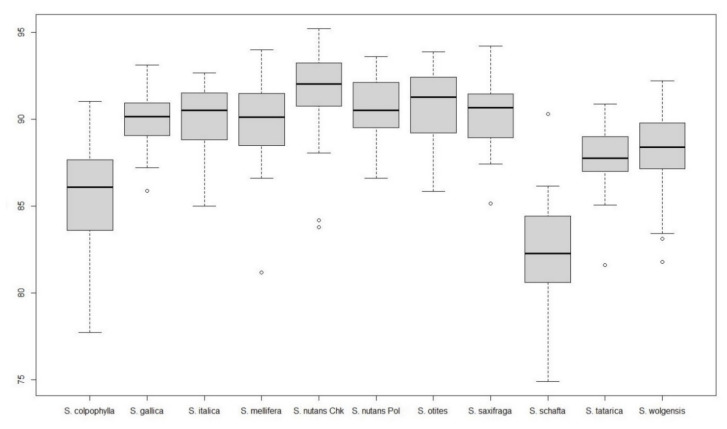
Box plot representing the values of *J* index with Model 1 for species of *S*. subg. *Silene*. Left to right: *S. colpophylla*, *S. gallica*, *S. italica*, *S. mellifera*, *S. nutans* Chk, *S. nutans* Pol, *S. otites*, *S. saxifraga*, *S. schafta*, *S. tatarica*, and *S. wolgensis*. Upper and lower limits of the discontinuous lines represent the maximum and minimum values not atypical (atypical values are outside, below the discontinuous lines). Lower and upper limits of the boxes represent respectively the first and third quartile. The thickened bar in the box is the median.

The images of *Silene* seeds resemble cardioids or modified cardioids: Figure 5 presents the silhouettes corresponding to 40 photographs of each species and Table 4 and Table 5 contain respectively the values of *J* index (percentage of similarity with the cardioid, Model 1) in the species of *S.* subg. *Behenanta* and *S.* subg. *Silene*. Figure 6 and Figure 7 contain the box plot representations for *J* index in species of *S.* subg. *Behenanta* and *S.* subg. *Silene*, respectively.

**Figure A3:**
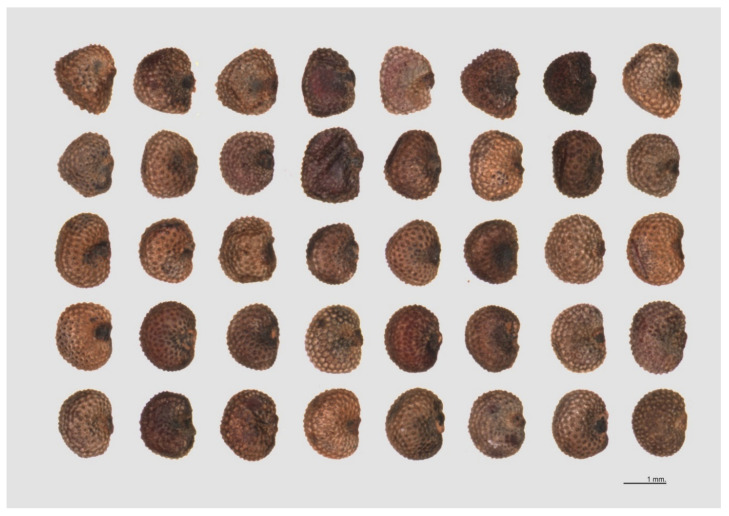
The seeds of *S. diclinis* used in this work ordered by increasing *J* index with Model 4. Bars represent 0.5 mm.

All the species in *S.* subg. *Behenantha* had mean values of *J* index superior to 90 (Table 4). The standard errors had the higher values in *S. diclinis*, *S. uniflora* and *S. zawadzkii* corresponding to notable variation in seed shape in these species (see Figure 6 and, for example, the 40 seeds of *S. diclinis* in Figure A3). The analysis includes two seed stocks of *S. dioica* collected in Poland and Czech Republic, and four of *S. latifolia*, three in Poland and one in Czech Republic. The values of *J* index obtained with the cardioid (Model 1) were similar in each pair of seed stocks of the same species with the only exception of *S. latifolia* Pol 1 and *S. latifolia* Chk.

Five species of *S.* subg. *Silene* gave mean values of *J* index below 90 (*S. colpophylla*, *S. mellifera*, *S. schafta*, *S. tatarica,* and *S. wolgensis*; Table 5). The seeds in *S. colpophylla*, *S. tatarica,* and *S. wolgensis* are markedly asymmetric (Figure 1), and thus different to a symmetric figure like the cardioid. The seeds of *S. schafta* have notable surface protuberances and many of them are also asymmetric (Figure 2), while *S. mellifera* seeds are variable in size and shape. The remaining species resemble the cardioid with values of *J* index comprised between 90.0 in *S. gallica* and 91.6 in *S. nutans*. The values of *J* index in the two seed stocks of *S. nutans* are similar.

#### 2.3.2. Comparison between Subgenera

The seeds of *S.* subg. *Behenantha* were larger than seeds of *S.* subg. *Silene* (Table 6). *S.* subg. *Silene* had displayed higher aspect ratio values. Mean circularity was similar in both subgenera while roundness and *J* index were higher in *S.* subg. *Behenantha*. Variation rate (standard deviation compared to mean values) was lower for aspect ratio, circularity and roundness when compared to the measurements related with length and area (area, perimeter, length, width), and still lower in *J* index.

#### 2.3.3. Similarity with Other Figures Related the Cardioid

**Figure 8:**
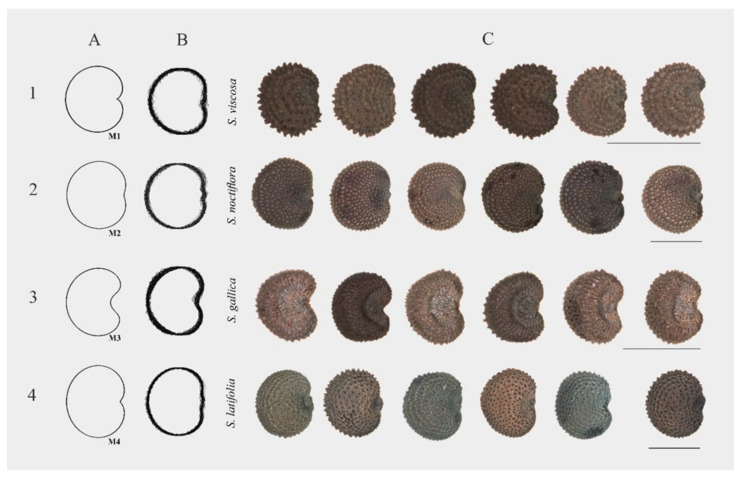
Comparison of seed shape in *Silene* species with geometric models. The first row shows the cardioid (Model 1; **1A**), the composed image of forty silhouettes (**1B**) and representative seeds of *S. viscosa* (*J* index with Model 1 = 92.6; **1C**). The second row shows the flattened cardioid (Model 2; **2A**), the composed image of forty silhouettes (**2B**) and representative seeds of *S. noctiflora* (*J* index with Model 2 = 94.4; **2C**). The third row contains the open cardioid (Model 3; **3A**), the silhouettes (**3B**) and seed images of *S. gallica* (*J* index with Model 3 = 90.4; **3C**), and the fourth row contains the flattened and elongated cardioid (Model 4; **4A**), silhouettes (**4B**) and images of *S. latifolia* (*J* index with Model 2 = 93.7; **4C**). Bars represent 1 mm.

The cardioid (Model 1) has already been tested in model plants and other species [34,35,36,37,38,39], and it was applied in general to all species tested in this work. The comparisons of the seed images with the cardioid gave good results (*J* index superior to 90) with many species in both subgenera, but our interest is to obtain models specific for particular species, i.e., that give high values with one species and low in the others. The observation of the composed images of the seed silhouettes (Figure 5) suggested that other cardioid-derived models could fit better the shape of some species. Models 2, 3 and 4 were specifically designed to increase values of *J* index in particular species. They were obtained by simple algebraic modifications of the cardioid equation (see Materials and Methods). Models 2 and 4 where designed considering those seeds whose ventral side is flatter than a cardioid, such as *S. diclinis, S. latifolia,* and *S. noctiflora*. Model 4 is slightly thinner than Model 2, and this can favor the resemblance to particular species, such as *S. diclinis*, while decreasing similarity to others, more rounded, such as *S. noctiflora*. Model 3 was designed based on the peculiar structure of the seeds of *S. gallica,* with a pronounced entry around the micropillar region. *J* index was calculated for the new models with the species resembling more to each of them. The results are shown in Figure 8 and Table 7 and Table 8.

**Figure 9:**
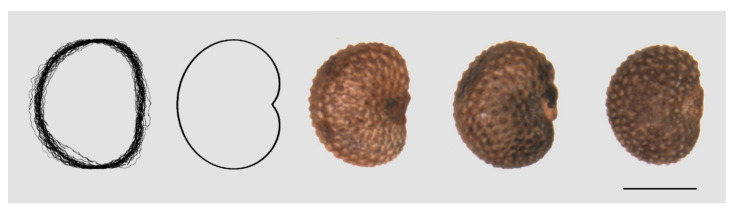
From left to right: The silhouettes of 40 images of *S. diclinis* seeds merged together in one image, Model 4 and three representative seeds of this species. Bar represents 1 mm.

Model 4 represents an improvement respect to Model 1 for the description and quantification of seed shape in *S. diclinis*. The value obtained with Model 4 in *S. diclinis* is remarkable considering that the seeds of this species present variable shapes, with some of them resembling the model (Figure 9) and others different from it (see the image composition in Figure A3). In addition, the values of *J* index with Model 4 also decreased for other species (see for example the values obtained with Model 4 with *S. noctiflora* in Table 7), this result adding value in favor of the specificity of this model for *S. diclinis*.

With Model 1 higher values of *J* index were obtained in *S. conica* than in *S. gallica* or *S. otites*, while the *J* index obtained with Model 3 was higher in *S. gallica.* From the value of 90 obtained with Model 1, the *J* index value increased in *S. gallica* to 90.4 with Model 3 (Table 8). Thus, Model 3 represents an improvement for the description and quantification of seed shape in *S. gallica*. Not only because the values of *J* index increased for this species, also because they decreased with other species.

### 2.4. Multivariate Analysis

**Figure 10:**
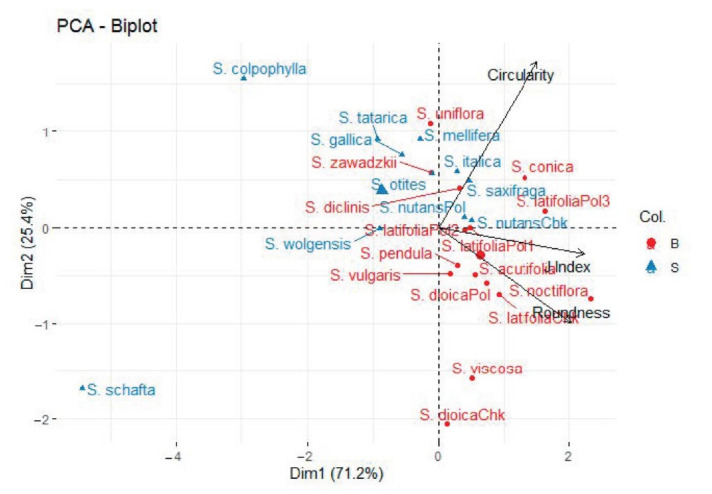
Principal Component Analysis showing the distribution of representative species belonging to *S*. subg. *Behenantha* (red) and *S*. subg *Silene* (blue) regarding geometrical models based on seed morphological data.

**Figure A4:**
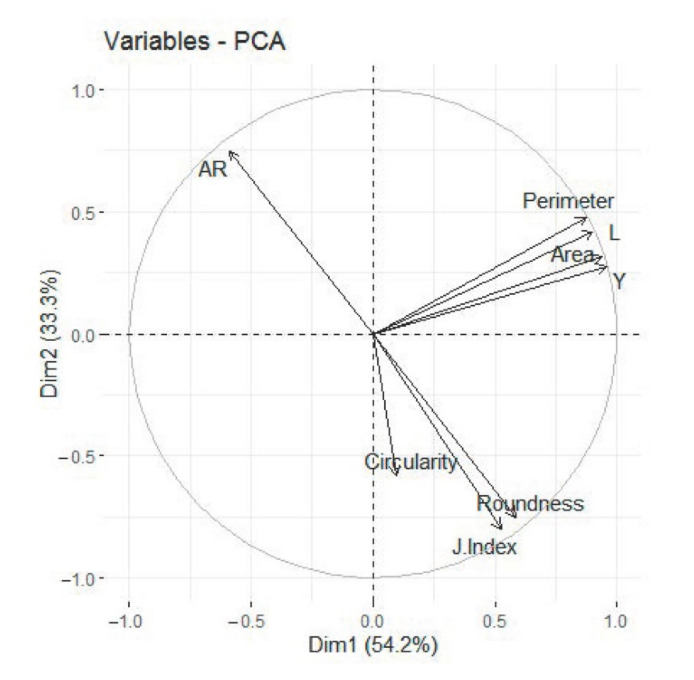
Principal component analysis with the distribution of all the variables measured. Variables cluster into two main groups in PC2, those from direct measurements are in the positive values of the ordinate axis and those fitting geometrical models are in the negative values of the ordinate axis.

A PCA with the distribution of all the variables clustered them into two main groups in dimension 2. Those from direct measurements are found in the positive values of the ordinate axis and those fitting geometrical models are found in the negative values of the ordinate axis (Figure A4). Geometrical models from complex calculations are more informative, and less influenced by the high levels of homoplasy. For this reason, a new PCA using only the variables belonging to geometrical models was performed. In Figure 10 we see the PCA graph with an explained variance of 96.7%. Red circles (with names in red color) represent species from *S.* subg. *Behenantha*, blue triangles (with names in blue color) represent species from *S.* subg. *Silene*. Variables corresponding to the geometrical models are represented in black.

In the dimension 2 of the PCA, *J* index and roundness are present together in the negative values of the ordinate axis. Regarding the different species, their mean distribution for *S.* subg. *Behenantha* and for *S.* subg *Silene* is opposite in both dimensions. In dimension 2, the same distribution is observed for *S.* subg. *Behenantha* and the variables Roundness and *J* index, while *S.* subg *Silene* and variable Circularity are found in the positive values of the ordinate axis. It is important to highlight that in the PC, those stocks from the same species share the same axis, including *S. latifolia*, which practically overlapped this distribution. According to the MANOVA (Table A1), we found significant differences when the analysis was applied according to the subgenera.

## 3. Discussion

Seed morphology in the genus *Silene* has been studied for decades as it provides important keys for the taxonomy and understanding the evolution of this genus [5,6,25,27,30,31]. In the genus *Silene*, seed morphological features like shape, structure of lateral face including macro and micromorphological traits have been useful for species identification and classification with consistent data for species in sections Conoimorphae, Melandriformes, and Sclerocalycinae [31].

We present here an original approach to seed morphology in *Silene* species based on the comparison of the seed images with geometric figures taken as models. This work includes data from twenty six seed stocks belonging to twenty one species of two subgenera, *S.* subg. *Behenantha* and *S.* subg. *Silene*, including the seeds of *S. diclinis*, an endangered species [10]. First, a general description based on the measurements of seed area, perimeter, length, width, aspect ratio, circularity, and roundness, is presented. Distinctive characters relevant for seed shape description include the texture of the seed surface, seed symmetry, presence of a conspicuous ridge and presence and characteristics of the dorsal face. These are useful for the identification of some species and may be associated with more general morphological patterns. Our main objective was to describe the shape of seeds based on the comparison of seed images with geometric figures as it was done in other species [32,33,34,35,36,37,38,39,40,41,42,43,44].

Geometrical models for the description of seed shape in *Silene* seeds have been used for the first time in this work and include the cardioid and three figures related to it. The fact that in the distribution of the different parameters, those belonging to geometrical models clustered together, supports the idea of using geometric figures as models for seed morphological analysis. The percentage of similarity of forty seed images with the cardioid (*J* index) has been calculated. *J* index values were more stable than the other measures considered (area, perimeter, length, width, aspect ratio, circularity and roundness) based on two arguments: First, the values of their standard deviations were low in relation to the means; and second, when *J* index was compared with the values of seed stocks from different origins belonging to the same species (*S. dioica*, *S. latifolia*) this values were generally held with a single exception. Our results indicate that the association of seed morphology with geometrical figures is very robust and could be used for classification purposes [43,44,45].

The mean values of *J* index were higher in *S.* subg. *Behenantha* than in *S.* subg. *Silene*. This being related with higher variation and more frequent presence of asymmetric seeds in the latter. In general, asymmetric seeds are expected to give lower values of *J* index with symmetric models. *J* index showed the same distribution with subgenus in both, PC1 and PC2. This confirms the observation of Rohrbach [25] (p.49) that seed shape offers the most reliable indicator for the differentiation of many species. Not only the distribution in the PCA showed differences, but also this result was supported by the MANOVA analysis, which showed statistical differences as well. This result indicates that values of a geometrical index (cardioid-shape in this case) may be useful to find differences in subgenera of *Silene*. Morphological traits have been usually applied for phylogenetic classification in plants [46,47]. On the other hand, *Silene* possess a high level of homoplasy in morphological characters, which leads to special difficulties in the phylogenetic interpretation [14]. As a matter of fact, the diagnostic characters of the recognized taxonomic groups of *Silene* significantly overlap, and they may be characterized by a set of combined morphological features especially related to the inflorescences, indumentum of the calyx and their morphology in flower and in fruit [14]. In our study, homoplasy is observed in *S. uniflora* and *S. zawadzkii* which do not follow the trend of subg. *Behenantha*. Several taxa from *S.* subg. *Behenantha* also show classification problems in phylogenetic analysis and specific morphological studies have been recommended [14]. For this reason, we suggest geometric indexes applied to seed morphology as a tool. Combined with molecular phylogenetic data, geometric indexes could be used for phylogenetic classification, as it has been proposed in other plant species of difficult classification [48].

*Silene latifolia* seeds showed a good adjustment to Models 2 and 4. This is of particular interest because sexual determination in this species is based on a system of remarkable similarity to the human XY pair, but where the Y chromosome is more susceptible to analysis [49]. This species is considered a model for the study of evolution and chromosome modification [50], and it may be of interest to evaluate diverse populations in this species for their morphology, as well as to correlate seed shape with chromosomic variations and genetic alterations. Model 4 describes seed shape in *S. diclinis*, one of the five endangered species of *Silene* in the Flora of the Iberian Peninsula. High similarity to Model 3 is associated to *S. gallica* with the presence of a ridge. It could be relevant to check if these two characteristics may coincide in other species of *Silene*, and check for the presence of seeds resembling Model 3 in other species.

The presented method allows the description of seed morphology based on geometric models. It is reproducible and useful to differentiate between seeds of different species providing a new approach for taxonomic studies of the genus *Silene*. With this basis the application of the adjectives reniform, circular and globular should be made with reserve. Reniform means kidney-shaped, but the kidney is not a geometric figure defined by an equation that can be objectively reproduced for comparison with other objects. In addition, a kidney, a circle or a globule refer to three dimensional figures and, in so far, there is no way to measure a three-dimensional shape in seeds. In comparison with elliptic Fourier analysis this method compares bi-dimensional seed shape with a figure of reference, thus the results are not purely numerical or statistical, but also testable directly by visual observation. A high value in *J* index means that the seeds of a species have a given shape, defined by the similarity to a model; and the difference detected between two species or varieties as the result of a statistical test means that there are morphological differences related with a different degree of similarity to a given model between the species or varieties tested. Thus, the results of a statistical test are compared directly with visual information given by the models.

Quantification of *J* index in a seed lot requires a certain degree of homogeneity in the sample under analysis, which may be difficult in seeds under stress conditions, stored for long time and for those belonging to endangered species, due to reduced seed availability, or absence in extreme cases [51]. In general, good adjustments to models were found in species with lower variation in shape. The case of *S. diclinis* is exceptional with a good adjustment to Model 4 associated with high diversity in shape. In the sample analyzed of this species there is a clear difference between seeds conforming to the model and others diverging from it. In the attempt to improve reproduction of this species it may be interesting to check for physiological differences associated with the morphological types, as well as to detect the relative frequency of morphological types in population studies. This opens up the possibility that the method here reported may be of help for the analysis and identification of different natural populations of this and other protected species. Perhaps this first indication of a certain morphological separation in *S. diclinis* could be indicating some separation of the genetic information of these populations that could be of interest for conservation purposes in the future.

Seed shape is closely related to those gene families playing a role in the proper morphological development, and changes in these genes greatly affect seed morphology [52]. Morphological seed parameters can be used to delimit genera and are also used together with sequencing data in phylogeny analyses [53,54,55]. Our results indicate a common morphological pattern conserved by seeds from subgenus *Behenantha* which shows some similarities to the phylogenetic gene tree analysis in *Silene* [48].

The method presented links seed morphology to functional traits through a geometrical approach. Not all the criteria used in Phylogeny have the same informative value. Individual morphological traits may be homoplastic which means very low informative value. On the other hand, a geometric model includes several traits which may overcome this problem. In addition, area, perimeter, length or width don’t provide information on shape. Circularity or roundness are more informative in this regard, but their usefulness depends on the similarity of the figure with a circle, being of scarce utility in low values. In the cases of a good adjustment to a model, *J* index provides valuable information in a single measurement and it may be, in consequence, useful in Phylogeny studies. Other analyses similar to the reported here for *Silene* species have revealed differences in species belonging to other families [38,39,40,41,42,43,44,45] and describe a morphological character that could support the taxonomic treatments based on molecular phylogenies of the groups considered.

The combination of different seed traits could be used as a tool for seed management in genebanks, avoiding in some cases more expensive and time-consuming analysis. Likewise, variations in seed morphology due to stress because of climatic change could be identified on those individual seeds that do not fall into the range of the geometrical index assigned to a concrete plant species. A correct evaluation of the different morphological indexes could be a suitable tool in phylogenetic analyses.

## 4. Materials and Methods

### 4.1. Species of Silene

We examined seeds of 26 seed stocks belonging to 21 species of *Silene* conserved in the plant collections of the Laboratory of Plant Ecology and Adaptation, University of Lodz (Poland) and the Botanic Garden of the University of Warsaw and laboratories of the Academy of Sciences of Brno (Czech Republic). Species are listed in Table 9 with an indication of their locations of origin (when known). Of them, three were annuals (*S. conica*, *S. noctiflora*, *S. pendula*), one annual or biennial (*S. gallica*), four biennials or perennials (*S. colpophylla*, *S. otites*, *S. viscosa*, *S. wolgensis*) and thirteen perennials [29,56,57,58,59,60,61,62,63,64,65] (see Table 9). *S. latifolia* is mentioned as being perennial and never annual by Bojňanský and Fargašová [29], but as being annual or short-lived perennial by efloras.org [56]. Species nomenclature was adopted after Oxelman et al. [8] and Plants of the world online [66]. For the classification of species as *S.* subg. *Behenanta* or *S.* subg. *Silene,* we followed Sileneae classification [67].

### 4.2. Seed Images

**Figure 11:**
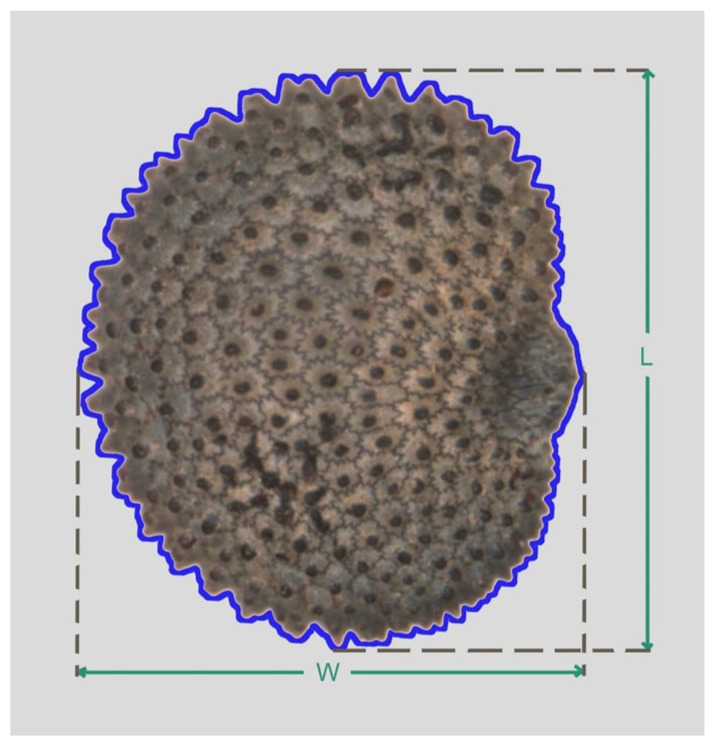
An image of *Silene* (*S. latifolia*). The perimeter (P) is indicated by the blue color. Length of the major axis (L) and length of the minor axis (W) are indicated. The hilium is included as a part of the seed in all measurements.

Photographs were taken with a Nikon Stereomicroscope Model SMZ1500 equipped with a camera Nikon DS-Fi1 of 5.24 megapixels. The seeds were oriented with the micropyle to the right (Figure 11). Composed images containing 40 seeds per accession were prepared with Corel Photo Paint and are stored in: https://zenodo.org/record/4057708#.X3LRpRRxeM8 and https://zenodo.org/record/4035649#.X3LRShRxeM8.

Confocal images were obtained with a Leica DM IRB TCS SP2 confocal microscope and are limited to a total of 13 species. Each figure contains the mean projection of a series of 20 images.

### 4.3. Surface Characteristics and other Structural Properties of Seeds

The presence and types of colliculae were evaluated in the stereomicroscopic images and by confocal microscopy. Other structural properties analyzed in the sets of stereomicroscopic images are: (1) seed asymmetry, (2) presence of ridges, and (3) pronounced dorsal surface (plane or concave).

### 4.4. General Morphological Description by Image Analysis

Photographs were used to obtain the area (A), perimeter (P), length of the major axis (L), length of the minor axis (W), aspect ratio (AR is the ratio L/W), circularity (C) and roundness (R). All the measurements are obtained with ImageJ program [68] by the conversion of pixel units to length or surface units (mm or mm^2^) using a ruler as a reference. The circularity index and roundness were calculated as described [69]. Circularity is the ratio (4π × A)/P^2^, while roundness is (4 × A)/πL^2^.

### 4.5. Comparison with Geometric Models: Calculation of the J Index

**Figure 12:**
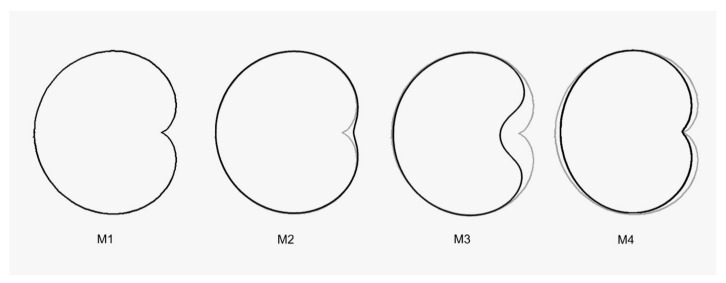
Models used in the geometric description of seeds from *Silene* species. Model 1, the cardioid curve, corresponding to Equation (1), was obtained from Mathematica [70]. Models 2 to 4 were obtained by the modification of Equation (1) searching for similarity with the outlines of particular seed images. Model 1 is superimposed with thin-line overlay in Models 2 to 4 to appreciate the differences.

A new approach to the morphological description of seed shape in *Silene* species is based in the comparison with geometric figures used as models. A set of four models is described and applied for the first time in this work. These are as follows (Figure 12):

Model 1: The cardioid curve is described by the equation:

(1) $$\left(x^{2}+y^{2}+ax{)}^{2}=a^{2}(x^{2}+y^{2}\right)$$

Model 2: A flattened cardioid, with a reduced discontinuity in the region of the hilium in relation to Model 1, is given by:

(2) $${\left(x^{2}+y^{2}+ax\right)}^{2}=b^{2}\left(x^{2}+y^{2}\right)$$

Model 3: An open cardioid is given by:

(3) $${\left(ax^{2}+ay^{2}+x\right)}^{2}=x^{2}+y^{2}+y/a$$

Model 4: A flattened and elongated cardioid, also with a reduced discontinuity in the region of the hilium but thinner, less rounded, than Model 2 (see Figure 12) is given by:

(4) $${\left(12ax^{2}+10ay^{2}+10x\right)}^{2}=130x^{2}+100y^{2}$$

In all four equations a and b are real positive parameters. Models 2 and 4 were selected visually by their similarity with the silhouettes of *S. latifolia* and *S. noctiflora*. Model 3 was selected for its similarity to *S. gallica*. Mathematica code for the models is available at: https://zenodo.org/record/4120172#.X5nooUeg-M9.

**Figure 13:**
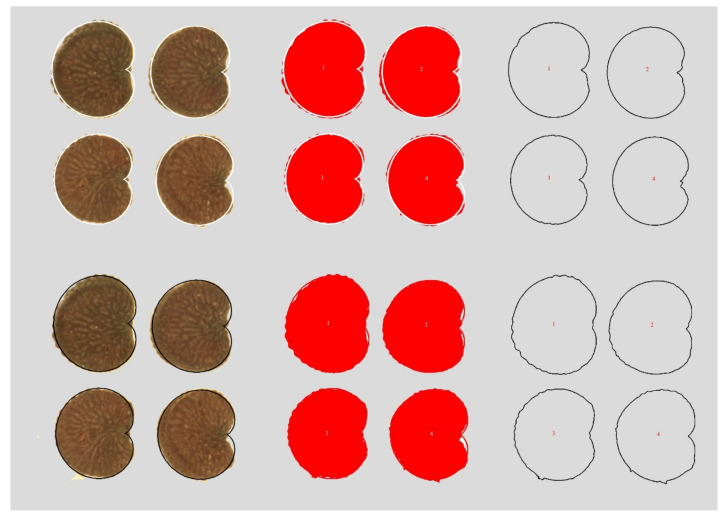
Representative samples of the composition of seed images and the geometric models used in the calculation of the *J* index. Four seed images are presented with the model superimposed in white (left, above) and the same images with the model in black (left, below). In the center (red colored), the corresponding images after modification in ImageJ (Image Type: 8 bit; adjust threshold). This way, the shared areas are observed and can be quantified as the larger areas in red (above), while total area is obtained as the total surface limited by the red limit (below). In the right, the four silhouettes above correspond to shared (S) and the four silhouettes below represent total area (T). The surfaces are observed and quantified with ImageJ. *J* index is the ratio S/Tx100.

Graphic compositions were done departing from one image containing forty seeds for each seed stock and subsequently elaborated with Corel PHOTO-PAINT X7. For the comparison of a group of seeds with the models and quantification of *J* index, the geometric figures used as models were superimposed to each seed image in the group of forty, searching a maximum adjustment between both shapes, the seeds and the model. An image scaled of the cardioid was adapted to the seed images and the percent of similarity between the image scaled of the cardioid and the seed image was estimated. Three graphic documents were kept for each composition: (1) A file in PSD format with the forty seeds and the geometric figure adapted to each of them, in which it is possible to make changes and corrections; (2) A file in JPG format with the geometric models in black, that served (later on) to obtain total area (T) with ImageJ, and (3) Another file in JPG format with the geometric models in white, that was used to obtain the values of area shared between the geometric figure and the seed image (S) in ImageJ. All the process of image composition with seeds and models was done in Corel PHOTO-PAINT X7, while area quantification was calculated in ImageJ. Figure 13 presents examples of the adjustment between seed images and the geometric models with indication of the areas measured for the calculation of the *J* index.

The images used are provided as Supplementary Files in Zenodo (https://zenodo.org/record/4057744; 4057740; 4057809; 4057810; 4020369; 4020382; 4057838; 4057831).

Seeds from all species were compared to Model 1; in addition, *S. latifolia* and *S. noctiflora* were compared to Models 2 and 4, because their seeds resemble more these models and in consequence, *J* index values obtained are higher. For the same reason, *S. conica*, *S. gallica,* and *S. otites* were compared to Model 3. To have good definitions of seed shape it is important to obtain high values of *J* index (high similarity with a given model means a good definition of a given shape). The areas corresponding to the two regions needed for the calculation of *J* index were obtained with ImageJ: the region that is common to the model and the seed image (“Shared” area, *S*) and the total region occupied by both areas, the seed and the model (“Total” area, *T*). The *J* index is defined by:

*J* index = (area *S*)/(area *T*) ×100

*S* is represented in Figure 13 as the area in the four silhouettes above (right), while *T* is the total area in the four silhouettes below (right). Note that *J* index is a measure of seed shape, not of its area. It ranges between 0 and 100 decreasing when the size of the not-shared region grows and equals 100 when the geometric model and the seed image areas coincide. High value of *J* index (high similarity with a given model) means a precise definition of seed shape for a particular species.

### 4.6. Statistical Analysis

The distribution of the raw data was skewed, and therefore had to be transformed to achieve homoscedasticity. One-way ANOVA was used to show significant differences between species for the measured variables, followed by Scheffé post-hoc tests to provide specific information on which means were significantly different from one another. This analysis was done with software IBM SPSS statistics v25 (SPSS 2017).

#### Multivariate Analysis

Multivariate analysis was done in R Studio, V.1.2.1335 [71]. Raw data was analyzed to check if the dataset was homoscedastic, what it means homogeneity of variance and normal distribution. A normalization of raw data to lower the weight of outliers prior to start with multivariate analysis was required and achieved using powerTransform function. A lambda value to transform the data was used according to mathematical procedure. This function uses the maximum likelihood-like approach of Box and Cox [72] to select a transformation of a univariate or multivariate response for normality. Principal Component Analysis is a procedure for dimension reduction used to see the total variation of variables and individuals in multidimensional data. The distribution of the species is based on their value for each parameter combined. This offers the possibility to gather species among them and to specific traits or, like in this analysis, to specific geometrical model indexes. In order to identify the statistical differences, a multivariate analysis of variance (MANOVA), a procedure for comparing multivariate sample means using the covariance, was applied. From the morphological description data, only those considered shape indexes (circularity index, roundness and *J* index) were used.

## 5. Conclusions

Images taken from well oriented seeds of *Silene* are described by comparison with a cardioid. *J* index is the percentage of similarity between the cardioid and the seed image. *J* index values are higher in species of *S.* subg. *Behenantha* than in *S.* subg. *Silene*.

Geometric figures derived from the cardioid by slight modifications in the corresponding algebraic equation adjust well to particular species of *Silene*. Thus, specific models are described for *S. gallica* as well as the endangered species *S. diclinis*.

The quantification of seed shape based in the comparison of *Silene* seeds with cardioid or cardioid-derived figures opens the way to new semi-automated methods of phenotyping. Variations in seed morphology due to stress in general, or climatic change, could be identified on those individual seeds that do not fall into the range of the geometrical index assigned to a concrete plant species. The asymmetry of the data does not affect the main conclusions and supports the hypothesis that in *Silene* the seeds of a population of the same species may follow different morphological patterns.

## Figures and Tables

**Table 1:** Mean values of the area (A), perimeter (P), length of the major axis (L), length of the minor axis (W), aspect ratio (AR is the ratio L/W), circularity (C) and roundness (R) in the seeds of species of *S*. subg. *Behenantha*. Values of A are given in mm^2^; P, L and W, in mm. Standard deviation values are given in parentheses. Superscript letters indicate the results of Scheffé test: The mean values marked with the same letter in each column do not differ significantly at *p* < 0.05. *n* is the number of seeds analyzed.

Species	*n*	A	P	L	W	AR	C	R
*S. acutifolia*	40	1.18 ^de^	4.55 ^de^	1.35 ^efg^	1.15 ^d^	1.18 ^cde^	0.72 ^de^	0.85 ^bcd^
		(0.14)	(0.32)	(0.08)	(0.07)	(0.06)	(0.03)	(0.04)
*S. conica*	40	0.52 ^i^	2.80 ^i^	0.89 ^j^	0.75 ^g^	1.18 ^cde^	0.83 ^a^	0.85 ^bcd^
		(0.03)	(0.08)	(0.03)	(0.02)	(0.04)	(0.02)	(0.03)
*S. diclinis*	40	1.73 ^b^	5.28 ^b^	1.65 ^b^	1.36 ^b^	1.21 ^bcd^	0.78 bc	0.83 ^cde^
		(0.14)	(0.24)	(0.07)	(0.06)	(0.05)	(0.03)	(0.04)
*S. dioica* Chk	40	1.30 ^c^	5.30 ^c^	1.42 ^cd^	1.21 ^c^	1.16 ^e^	0.59 f	0.86 ^b^
		(0.17)	(0.59)	(0.10)	(0.09)	(0.04)	(0.08)	(0.03)
*S. dioica* Pol	40	0.94 ^h^	4.07 ^h^	1.20 ^i^	1.02 ^f^	1.18 ^de^	0.71 ^de^	0.85 ^bc^
		(0.12)	(0.37)	(0.08)	(0.06)	(0.05)	(0.05)	(0.03)
*S. latifolia* Chk	40	1.15 ^gh^	4.52 ^gh^	1.33 ^hi^	1.12 ^f^	1.18 ^cde^	0.71 ^ab^	0.85 ^bcd^
		(0.14)	(0.43)	(0.09)	(0.07)	(0.05)	(0.06)	(0.03)
*S. latifolia* Pol 1	40	1.07 ^fg^	4.29 ^fg^	1.29 g^h^	1.06 ^ef^	1.21 ^abc^	0.74 ^d^	0.82 ^def^
		(0.16)	(0.42)	(0.09)	(0.09)	(0.03)	(0.04)	(0.02)
*S. latifolia* Pol 2	40	1.26 ^cd^	4.67 ^cd^	1.41 ^cde^	1.14 ^d^	1.22 ^ab^	0.73 ^d^	0.82 ^ef^
		(0.19)	(0.33)	(0.10)	(0.08)	(0.03)	(0.05)	(0.02)
*S. latifolia* Pol 3	40	1.02 ^ef^	3.97 ^ef^	1.23 ^fg^	1.04 ^d^	1.18 ^de^	0.80 ^de^	0.85 ^bc^
		(0.18)	(0.31)	(0.11)	(0.09)	(0.03)	(0.04)	(0.03)
*S. noctiflora*	40	1.28 ^cd^	4.55 ^cd^	1.36 ^def^	1.21 ^c^	1.12 ^f^	0.78 ^c^	0.90 ^a^
		(0.11)	(0.21)	(0.06)	(0.05)	(0.04)	(0.02)	(0.03)
*S. pendula*	40	1.14 ^ef^	4.48 ^ef^	1.34 ^efg^	1.11 ^de^	1.20 ^bcde^	0.71 ^de^	0.84 ^bcde^
		(0.10)	(0.23)	(0.07)	(0.07)	(0.08)	(0.03)	(0.05)
*S. uniflora*	40	1.31 ^c^	4.51 ^c^	1.43 ^c^	1.16 ^cd^	1.25 ^a^	0.80 a^b^	0.80 ^f^
		(0.13)	(0.23)	(0.09)	(0.06)	(0.07)	(0.01)	(0.04)
*S. viscosa*	40	0.51 ^i^	3.19 ^i^	0.89 ^j^	0.75 ^g^	1.17 ^de^	0.63 ^f^	0.85 ^bc^
		(0.05)	(0.18)	(0.05)	(0.04)	(0.04)	(0.04)	(0.03)
*S. vulgaris*	40	1.20 ^cde^	4.67 ^cde^	1.37 ^cdef^	1.14 ^d^	1.20 ^bcd^	0.69 ^e^	0.83 ^cde^
		(0.15)	(0.36)	(0.09)	(0.08)	(0.06)	(0.04)	(0.04)
*S. zawadzkii*	40	2.02 ^a^	5.74 ^a^	1.79 ^a^	1.46 ^a^	1.23 ^ab^	0.77 ^c^	0.81 ^ef^
		(0.24)	(0.36)	(0.10)	(0.08)	(0.06)	(0.02)	(0.04)

**Table 2:** Mean values of the area (A), perimeter (P), length of the major axis (L), length of the minor axis (W), aspect ratio (AR is the ratio L/W), circularity (C) and roundness (R) in the seeds of species of *S*. subg. *Silene*. Values of A are given in mm^2^; P, L, and W, in mm. Standard deviation values are given in parentheses. Superscript letters indicate the results of Scheffé test: the mean values marked with the same letter in each column do not differ significantly at *p* < 0.05. *n* is the number of seeds analyzed.

Species	*n*	A	P	L	W	AR	C	R
*S. colpophylla*	40	0.68 ^c^	3.44 ^de^	1.08 ^c^	0.82 ^d^	1.35 ^a^	0.72 ^e^	0.74 ^c^
		(0.07)	(0.18)	(0.05)	(0.06)	(0.08)	(0.02)	(0.04)
*S. gallica*	40	0.53 ^d^	2.97 ^e^	0.93 ^e^	0.74 ^f^	1.26 ^b^	0.76 ^cd^	0.79 ^c^
		(0.05)	(0.17)	(0.05)	(0.04)	(0.05)	(0.03)	(0.03)
*S. italica*	40	0.65 ^c^	3.21 ^e^	1.01 ^d^	0.83 ^d^	1.21 ^cd^	0.79 ^a^	0.83 ^ab^
		(0.07)	(0.16)	(0.05)	(0.05)	(0.07)	(0.01)	(0.05)
*S. mellifera*	40	0.99 ^a^	3.95 ^c^	1.25 ^ab^	1.01 ^b^	1.25 ^bc^	0.79 ^ab^	0.80 ^bc^
		(0.21)	(0.40)	(0.13)	(0.12)	(0.07)	(0.03)	(0.04)
*S. nutans* Chk	40	1.06 ^a^	4.25 ^b^	1.3 ^a^	1.06 ^a^	1.19 ^cd^	0.73 ^d^	0.81 ^ab^
		(0.12)	(0.24)	(0.05)	(0.08)	(0.06)	(0.04)	(0.05)
*S. nutans* Pol	40	1.07 ^a^	4.22 ^b^	1.29 ^a^	1.07 ^a^	1.21 ^cd^	0.75 ^d^	0.83 ^ab^
		(0.13)	(0.23)	(0.07)	(0.07)	(0.05)	(0.03)	(0.04)
*S. otites*	40	0.41 ^d^	2.60 ^f^	0.81 ^e^	0.66 ^f^	1.24 ^bcd^	0.76 ^cd^	0.81 ^abc^
		(0.06)	(0.20)	(0.07)	(0.05)	(0.07)	(0.02)	(0.05)
*S. saxifraga*	40	0.78 ^b^	3.52 ^d^	1.11 ^c^	0.92 ^c^	1.20 ^d^	0.79 ^a^	0.84 ^a^
		(0.11)	(0.27)	(0.08)	(0.07)	(0.07)	(0.02)	(0.04)
*S. schafta*	40	0.82 ^b^	4.79 ^a^	1.22 ^b^	0.93 ^c^	1.34 ^a^	0.45 ^h^	0.75 ^c^
		(0.11)	(0.59)	(0.09)	(0.07)	(0.06)	(0.06)	(0.03)
*S. tatarica*	40	0.54 ^d^	2.96 ^e^	0.93 ^e^	0.76 ^ef^	1.25 ^bc^	0.77 ^bc^	0.80 ^bc^
		(0.05)	(0.14)	(0.04)	(0.04)	(0.06)	(0.02)	(0.04)
*S. wolgensis*	40	0.57 ^d^	3.21 ^e^	0.97 ^de^	0.80 ^de^	1.23 ^bcd^	0.70 ^f^	0.82 ^abc^
		(0.05)	(0.18)	(0.05)	(0.04)	(0.07)	(0.03)	(0.05)

**Table 3:** Summary of the variability in the morphological characteristics of the species of *Silene* studied. A minus sign (-) indicates the absence (or predominance of absence) and a plus sign (+) the presence (predominance of presence) of the character indicated for a given species. An asterisk indicates the species in which only few seeds were observed with ridge. When two equal signs coincide in two opposite characters it means that none of them is predominant.

Subg.	Species	Symmetry	Dorsal Face		Ridge	Hilium	
			Plane	Canaliculate		Almost None	Almost All
*Behenantha*	*S. acutifolia* Link ex Rohrb.	** - **	+	-	-	+	-
	*S. conica* L.	+	-	+	-	+	-
	*S. diclinis* (Lag.) M.Laínz	+	-	-	-	-	-
	*S. dioica* (L.) Clairv.	+	-	-	-	-	+
	*S. latifolia* Poir.	+	-	-	-	-	-
	*S. noctiflora* L.	+	-	-	-	-	+
	*S. pendula* L.	+	+	-	-	-	-
	*S. uniflora* Roth	+	+	-	-*	-	-
	*S. viscosa* (L.) Pers.	+	-	-	-	-	-
	*S. vulgaris* (Moench) Garcke	+	+	-	-	-	+
	*S. zawadzkii* Herbich	-	+	-	-*	-	+
*Silene*	*S. colpophylla Wrigley*	-	+	+	-	+	-
	*S. gallica* L.	+	-	+	+	+	-
	*S. italica* (L.) Pers.	+	+	+	-	+	-
	*S. mellifera* Boiss. and Reut.	-	+	-	-	+	-
	*S. nutans* L.	+	+	-	-*	-	-
	*S. otites* (L.) Wibel	-	+	+	-	-	-
	*S. saxifraga* L.	-	+	+	-	-	-
	*S. schafta* S.G.Gmel.	-	+	-	-	+	-
	*S. tatarica* (L.) Pers.	-	+	+	-	+	-
	*S. wolgensis* (Hornem.) Otth	-	+	+	-	+	-

**Table 4:** Values of *J* index with the cardioid as a model in *S*. subg. *Behenantha*. Superscript letters indicate the results of Scheffé test: The mean values marked with the same letter in each column do not differ significantly at *p* < 0.05. *n* is the number of seeds analyzed.

Species	*n*	*J* Index (Cardioid)	Min	Max	Standard Dev.
*S. acutifolia*	40	91.2 ^bcdef^	86	94.1	1.84
*S. conica*	40	92.1 ^bcd^	89	94.6	1.28
*S. diclinis*	40	90.3 ^ef^	83	93.6	2.63
*S. dioica* Chk	40	91.5 ^bcde^	88	94.1	1.18
*S. dioica* Pol	40	91.7 ^bcde^	85	95.8	1.34
*S. latifolia* Chk	40	92.5 ^a^	83	93.5	1.97
*S. latifolia* Pol 1	40	92.2 ^bcd^	89	94	0.88
*S. latifolia* Pol 2	40	92.4 ^abc^	91	95.2	1.18
*S. latifolia* Pol 2	40	93.5 ^abc^	87	95.8	1.14
*S. noctiflora*	40	93.6 ^a^	82	93.4	1.09
*S. pendula*	40	90.8 ^def^	89	94.5	2.1
*S. uniflora*	40	90.3 ^ef^	83	94.1	2.66
*S. viscosa*	40	92.6 ^ab^	83	94.5	1.33
*S. vulgaris*	40	91.18 ^cdef^	83	94	2.15
*S. zawadskii*	40	90.02 ^f^	83	94.5	2.57

**Table 5:** Values of *J* index with the cardioid as a model in *Silene* species (subg. *Silene*). Superscript letters indicate the results of Scheffé test: the mean values marked with the same letter in each column do not differ significantly at *p* < 0.05. *n* is the number of seeds analyzed.

Species	*n*	*J* Index (Cardioid)	Min	Max	Standard Dev.
*S. colpophylla*	40	85.6 ^e^	77.7	91.1	2.96
*S. gallica*	40	90.0 ^b^	85.9	93.1	1.51
*S. italica*	40	90.1 ^ab^	85	92.7	1.74
*S. mellifera*	40	89.8 ^bc^	81.2	94	2.31
*S. nutans* Chk	40	91.6 ^a^	83.7	95.2	2.43
*S. nutans* Pol	40	91.6 ^a^	83.8	94.9	2.46
*S. otites*	40	90.7 ^ab^	85.9	93.9	2.11
*S. saxifraga*	40	90.3 ^ab^	85.2	94.2	1.73
*S. schafta*	40	82.3 ^f^	74.9	90.3	2.88
*S. tatarica*	40	87.8 ^d^	81.6	90.9	1.64
*S. wolgensis*	40	88.3 ^cd^	81.8	92.2	2.28

**Table 6:** Comparison between subgenera of the mean values representative of size and shape: Area (A), perimeter (P), length of the major axis (L), length of the minor axis (W), aspect ratio (AR is the ratio L/W), circularity (C), roundness (R) and percent similarity with the cardioid (*J* index M1). Values of A are given in mm^2^; P, L, and W, in mm. Standard deviation values are given in parentheses. Superscript letters indicate the results of Scheffé test: The mean values marked with the same letter in each column do not differ significantly at *p* < 0.05. *n* is the number of seeds analyzed.

	*n*	A	P	L	W	AR	C	R	*J*I M1
*S*. subg *Behenantha*	600	1.18 ^a^ (0.4)	4.44 ^a^ (0.8)	1.33 ^a^ (0.24)	1.11 ^a^ (0.19)	1.19 ^a^ (0.06)	0.73 ^a^ (0.07)	0.84 ^a^ (0.05)	91.8 ^a^ (2.09)
*S*. subg. *Silene*	400	0.7 ^b^ (0.22)	3.5 ^b^ (0.69)	1.07 ^b^ (0.17)	0.86 ^b^ (0.13)	1.25 ^b^ (0.08)	0.73 ^a^ (0.1)	0.8 ^b^ (0.05)	88.7 ^b^ (3.4)

**Table 7:** Comparison between values of *J* index with Model 1 (M1; cardioid), Models 2 and 4 in seed images of *S. diclinis*, *S. latifolia* and *S.noctiflora*. Standard deviation values are given in parentheses. Superscript letters indicate the results of Scheffé test: The mean values marked with the same letter in each column do not differ significantly at *p* < 0.1 (*S. diclinis*), or at *p* < 0.05 (*S. latifolia* and *S. noctiflora*)*. n* is the number of seeds analyzed.

*J* Index Values	*S. diclinis* (*n* = 40)	*S. latifolia* (*n* = 160)	*S.noctiflora* (*n* = 40)
M1	90.3 ^c^ (2.66)	92.6 ^b^ (1.46)	93.6 ^a^ (1.11)
M2	91.2 ^c^ (2.18)	93.0 ^b^ (1.68)	94.4 ^a^ (1.14)
M4	91.5 ^b^ (2.42)	92.5 ^a^ (1.97)	89.6 ^c^ (2.53)

**Table 8:** Values of *J* index with Model 1 and Model 3 in *S. conica, S. gallica,* and *S. otites.* Superscript letters indicate the results of Scheffé test: The mean values marked with the same letter in each column do not differ significantly at *p* < 0.05 (*S. conica* and *S. otites*) or *p* < 0.16 (*S. gallica*)*. n* is the number of seeds analyzed.

*J* Index Values	*S. conica* (*n* = 40)	*S. gallica* (*n* = 40)	*S. otites* (*n* = 40)
*M1*	92.1 ^a^ (1.29)	90.0 ^b^ (1.52)	90.7 ^b^ (2.13)
*M3*	86.2 ^c^ (2.22)	90.4 ^a^ (1.01)	88.8 ^b^ (2.48)

**Table 9:** List of species described, indicating the origin of the seeds.

Species	Lab.	Origin	Annual, Biannual or Perennial
*S. acutifolia* Link ex Rohrb.	CzR	u (unknown)	P [57]
*S. colpophylla* Wrigley	CzR	France	B,P [58]
*S. conica* L.	CzR	Germany	A [59]
*S. diclinis* (Lag.) M.Laínz	CzR	Pla de Mora (Spain)	P [60]
*S. dioica* (L.) Clairv.	CzR/Pol	Tišnov (CzR)/u	P [29,58]
*S. gallica* L.	CzR	u	AB [29,61]
*S. italica* (L.) Pers.	CzR	u	BP [29,61]
*S. latifolia* Poir.	CzR/Pol (3 seed stocks)	Panenská Rozsíčka (CzR)/Dubidze(Pol)	P [29,56,61]
*S. mellifera* Boiss. and Reut.	Pol	u	BP [62]
*S. noctiflora* L.	CzR	Kuřim (CzR)	A [29,61]
*S. nutans* L.	CzR/Pol	u	P [29,61]
*S. otites* (L.) Wibel	CzR	Rohatec (CzR)	P [58,63]
*S. pendula* L.	CzR	u	A [29]
*S. saxifraga* L.	CzR	u	P [29]
*S. schafta* S.G.Gmel.	CzR	u	P [29]
*S. tatarica* (L.) Pers.	CzR	u	P [29,61]
*S. uniflora* Roth	Pol	u	P [64]
*S. viscosa* (L.) Pers.	CzR	Rohatec (CzR)	BP [61]
*S. vulgaris* (Moench) Garcke	CzR	Lomnička (CzR)	P [29,61,65]
*S. wolgensis* (Hornem.) Otth	CzR	Bashkortostan (RU)	B [29]
*S. zawadzkii* Herbich	CzR	u	P

## Supplementary Materials

The images containing the original photographs of seeds (40 seeds per image) are available online: https://zenodo.org/record/4057708#.X3LRpRRxeM8 and https://zenodo.org/record/4035649#.X3LRShRxeM8. The compositions of seed photographs with the cardioid or cardioid-derived figures used for the calculations of *J* index are available online: (https://zenodo.org/record/4057744; 4057740; 4057809; 4057810; 4020369; 4020382; 4057838; 4057831).

## Appendix A

**Table A1:** Multivariate analysis of variance (MANOVA) from seed morphological data. Statistically significant differences can be found according to subgenus.

MANOVA (Multi Analysis of Variance)						
	Df	Pillai	Approx. F num.	Df	Den DF	Pr (>F)
**(Intercept)**	1	1	1,440,064	3	21	<2.2 × 10^−16^ ***
**Subgenus**	1	0.42024	5	3	21	0.008473 **
**Residuals**	23					

** *p* < 0.01, *** *p* < 0.001.
